# ﻿*Xeniakonohana* sp. nov. (Cnidaria, Octocorallia, Alcyonacea), a new soft coral species in the family Xeniidae from Miyazaki, Japan

**DOI:** 10.3897/zookeys.1085.77924

**Published:** 2022-02-03

**Authors:** Tatsuki Koido, Yukimitsu Imahara, Hironobu Fukami

**Affiliations:** 1 Interdisciplinary Graduate School of Agriculture and Engineering, University of Miyazaki, 1–1 Gakuen-kibanadai-nishi, Miyazaki, Miyazaki 889–2192, Japan University of Miyazaki Miyazaki Japan; 2 Kuroshio Biological Research Foundation, 560 Nishidomari, Otsuki, Kochi 788–0333, Japan Kuroshio Biological Research Foundation Otsuki Japan; 3 Octocoral Research Laboratory, 300–11 Kire, Wakayama, 640–0351, Japan Octocoral Research Laboratory Kire Japan; 4 Geological Survey of Japan, National Institute of Advanced Industrial Science and Technology, 1-1-3 Higashi, Tsukuba, Ibaraki 305–8567, Japan Geological Survey of Japan, National Institute of Advanced Industrial Science and Technology Tsukuba Japan; 5 Department of Marine Biology and Environmental Sciences, Faculty of Agriculture, University of Miyazaki, 1–1 Gakuen-kibanadai-nishi, Miyazaki, Miyazaki 889–2192, Japan Miyazaki University Miyazaki Japan

**Keywords:** Alcyonacea, Cnidaria, Miyazaki, new species, *Xenia*, Xeniidae

## Abstract

A new soft coral species, *Xeniakonohana***sp. nov.** (Alcyonacea, Xeniidae), is described from Miyazaki in the warm-temperate region of Japan. This new species has conspicuous and unique spindle sclerites in addition to the simple ellipsoid platelet-shaped sclerites typically found in the genus *Xenia*. These unique spindles are a specific key morphological characteristic for this new species and for differentiating this species among congeneric species.

## ﻿Introduction

Species of the family Xeniidae are known as pioneers in tropical coral reefs (Benayahu and Loya 1987), playing an important role for ecological succession in coral reefs. Therefore, knowing how many species of Xeniidae exist, and the range of species diversity will be useful for understanding the coral reef ecosystem.

For species or genus identification of alcyonacean soft corals including xeniids, the shape and arrangement of sclerites are used as key characteristics. Xeniids typically produce minute platelets or corpuscle-like sclerites without tubercular differences among species and genera under light microscopy (Fabricius and Alderslade 2001). The microstructure of sclerites has been shown to be an important character at the genus level of the family Xeniidae. Recently, the type specimens of 21 species in the genus *Xenia* were rechecked and re-described using sclerite microstructure (Halász et al. 2019). Thus, observation of sclerite microstructure is taxonomically useful for species delimitation, at least in some species of *Xenia*.

The genus *Xenia* presently includes 49 valid species (Cordeiro et al. 2021). This genus is characterized by platelet-shaped sclerites with surface microstructure composed of calcite dendritic and sinuous rods (Alderslade 2001; Halász et al. 2019). Koido et al. (2019) reported an undescribed species belonging to *Xenia* (reported as *Xenia* sp. 1) from Oshima Island, Miyazaki, in the warm-temperate region (non-coral reef region) of Japan. This previous work emphasized the high species diversity of Xeniidae in Miyazaki, Japan. This study provides a description of this previously undescribed species (*Xenia* sp. 1) as *Xeniakonohana* sp. nov., a new species in the genus.

## ﻿Materials and methods

**Figure 1.**
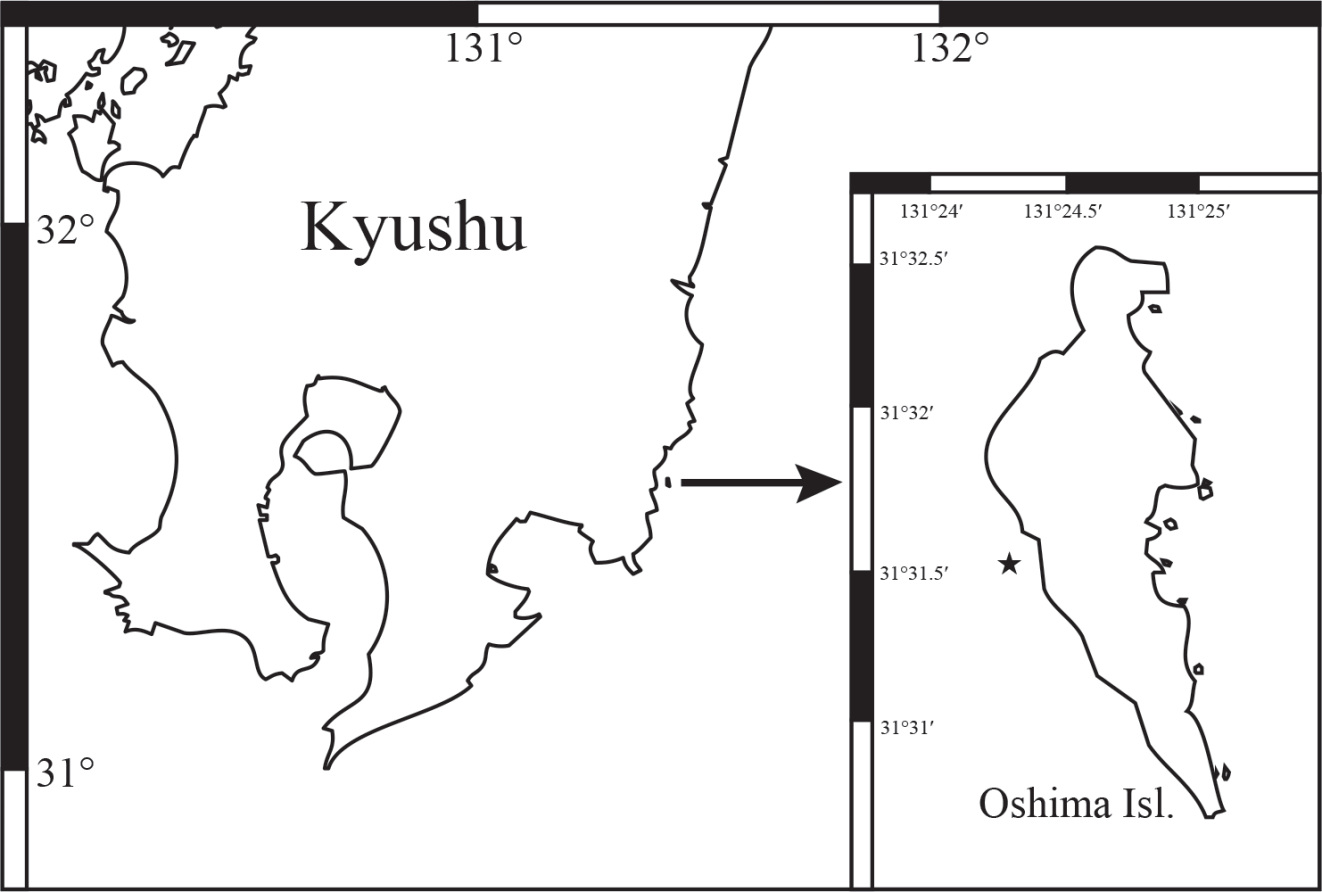
Collection sites of *Xeniakonohana* sp. nov. in Miyazaki, Japan.

All specimens were collected around Oshima Island (31°31.35'N, 131°24.27'E) (Fig. 1), Miyazaki, Japan, by SCUBA diving and snorkeling. A small piece of tissue (5–10 mm) from each specimen was used for molecular analyses and the remainder was preserved in 99% ethanol for morphological analyses as reported by Koido et al. (2019).

Specimens were previously deposited in Miyazaki University, Fisheries Sciences (MUFS) but were subsequently transferred and deposited at the Kuroshio Biological Research Foundation, Kochi, Japan (KBF) in the octocoral collection (OA). Morphological characteristics examined under a stereomicroscope included colony height, length and width of the stalk, presence of branches, length and width of polyps, length and width of tentacles, length and width of pinnules, number of rows of pinnules, and number of pinnules in the aboral row. Sclerites from polyps, and ones from the surface and interior of both stalk and branches of each specimen were examined. Sclerite shape, size, and microstructure were examined with light microscopy and scanning electron microscope (SEM) (HITACHI S-4800 and JEOL JSM-6500F).

### ﻿DNA extraction, amplification, and sequencing

Tissue samples were kept in CHAOS solution for at least a week to dissolve proteins at room temperature as reported by Koido et al. (2019). Total DNA was extracted from CHAOS solutions by conventional phenol/chloroform extraction. The phylogenetic position of *X.konohana* sp. nov. was inferred using three mitochondrial markers (*ND2*, *mtMutS*, *COI*) (16S647F: 5’-ACA CAG CTC GGT TTC TAT CTA CCA-3’; ND21418R: 5’ -ACA TCG GGA GCC CAC ATA-3’, ND42625F: 5’-TAC GTG GYA CAA TTG CTG-3’, Mut-3458R: 5’-TSG AGC AAA AGC CAC TCC-3’, COII8068F: 5’-CCA TAA CAG GAC TAG CAG CAT C-3’, HC02198: 5’-TAA ACT TCA GGG TGA CCA AAA AAT CA-3’) and a nuclear marker (*28S*) (28S-Far: 5’-CAC GAG ACC GAT AGC GAA CAA GTA-3’, 28S-Rar: 5’-TCA TTT CGA CCC TAA GAC CTC-3’). PCR reactions for all four markers used 1 μL of DNA solution, 1.6 μL of 2.5 mM dNTP Mixture, 2 μL of 10X Ex Taq buffer, 2 μL of each primer (10 mM), 0.08 μL Ex taq (TaKaRa), and 11.32 μL of sterile distilled water. Amplification of these markers used a GeneQ PCR Thermal Cycler with the following thermal profile; 35 cycles of 90 sec at 94 °C, 60 sec at 58 °C, and 60 sec at 72 °C. Amplicons were checked on 1% agarose gel electrophoresis. All PCR products were treated to remove excess primers and dNTP using Exonuclease I (TaKaRa) and Shrimp Alkaline Phosphatase (TaKaRa). DNA sequences were determined by ABI3000 using a research contract service (Ltd. FASMAC). DNA sequences of 709 bases for *mtMutS*, 804 for *COI*, 773 for *28S* rDNA, and 673 for *ND2* were obtained in this study. DNA sequences for *mtMutS*, *COI*, and *28S* were combined and analyzed because concatenated DNA sequences using these markers have been recently used for the molecular phylogenetic analyses in the Xeniidae (McFadden et al. 2019; Halász et al. 2019), while sequences for ND2 were analyzed alone because of restricted number of sequences available (McFadden et al. 2006; McFadden and Ofwegen 2012; McFadden et al. 2014b; McFadden et al. 2017). As outgroups for both analyses, we used *Paralemnaliathyrsoides* (Ehrenberg, 1834) (family Nephtheidae), *Rhytismafulvum* (Forskål, 1775) (family Alcyoniidae) and *Coelogorgiapalmosa* Milne Edwards & Haime, 1857 (family Coelogorgiidae), which are all known to be closely related to the Xeniidae (Halász et al. 2019). MEGA6 (Tamura et al. 2013) was used to select appropriate models (T92+G model for the concatenated DNA sequences, including *mtMutS*, *COI*, and *28S*, and T92 model for *ND2*) for maximum likelihood (ML) method and to reconstruct the ML phylogenetic trees with 1000 bootstrap replicates. In Bayesian analysis, the concatenated alignment data was treated as a separate data partition with different models of evolution applied to each of the mitochondrial (*mtMutS* and *COI*: HKY+G) and nuclear (*28S*: GTR+G) markers. MrBayes v. 3.2.1 (Ronquist et al. 2012) was run for 50,000,000 generations (until standard deviation of split partitions < 0.01) with a burn-in of 25% and default Metropolis coupling parameters. For phylogenetic analyses, recently published data for three markers (*mtMutS*, *COI*, and *28S*) from the Xeniidae were also added (Table 1).

**Table 1.** List of specimens of the family Xeniidae examined in this study and accession numbers for *28S*, *mtMutS*, *COI* and *ND2* markers. The origin of the accession number is shown by asterisk (s) in the reference list for each line if more than one reference exists.

Species	Specimen Catalog #	GenBank accession number				References
		*28S*	*mtMutS*	*COI*	*ND2*	
*Xeniakonohana* sp. nov.	KBF-OA–00092	LC656679*	LC656674*	LC656676*	LC467035**	*This study
						**Koido et al. 2019
*Xeniakonohana* sp. nov.	KBF-OA–00093	LC656680*	LC656673*	LC656677*	LC467036**	*This study,
						**Koido et al. 2019
*Xeniakonohana* sp. nov.	KBF-OA–00094	LC656681*	LC656675*	LC656678*	LC467037**	*This study,
						**Koido et al. 2019
*Antheliaglauca*	ZMTAU CO34183	JX203753*	JX203812*	GQ342460**	–	*McFadden and Ofwegen 2012, **Brockman and McFadden 2012
*Asterospicularialaurae*	CSM-OCDN8971L	KM201433	KM201452	KM201458	–	Janes et al. 2014
*Asterospiculariarandalli*	RMNH:Coel. 41521	KF915316	KF915556	KF955019	–	McFadden et al. 2014a
*Heteroxeniamindorensis*	CAS:IZ:184566	KJ511300	KJ511339	KJ511379	KJ511421	McFadden et al. 2014b
*Heteroxeniamindorensis*	CAS:IZ:184574	KJ511381	KJ511341	KJ511302	KJ511423	McFadden et al. 2014b
*Ovabundaainex*	ZMTAU:36785	KY442364	KY442323	KY442342	KY442395	McFadden et al. 2017
*Ovabundaainex*	ZMTAU:36786	KY442365	KY442324	KY442343	KY442396	McFadden et al. 2017
*Ovabundaandamanensis*	PMBC:11861	KM201440	KM201455	KM201461	–	Janes et al. 2014
*Ovabundaandamanensis*	PMBC:11862	KM201439	KM201454	KM201460	–	Janes et al. 2014
*Ovabundabiseriata*	ZMTAU:34876	KY442376	KY442330	KY442349	KY442405	McFadden et al. 2017
*Ovabundabiseriata*	ZMTAU:34881	KY442378	KY442332	KY442351	KY442407	McFadden et al. 2017
*Ovabundabiseriata*	ZMTAU:34882	KY442379	KY442333	KY442352	KY442408	McFadden et al. 2017
*Ovabundafaraunenesis*	ZMTAU:CO 34051	KJ511306**	GU356029*	GU356006*	KJ511427**	*McFadden et al. 2011, **McFadden et al. 2014b
*Ovabundafaraunenesis*	ZMTAU:34884	KY442380	KY442334	KY442353	KY442412	McFadden et al. 2017
*Ovabundafaraunenesis*	ZMTAU:34886	KY442381	KY442335	KY442354	KY442413	McFadden et al. 2017
*Ovabundaimpulsatilla*	ZMTAU:34571	KY442374	KY442328	KY442347	KY442418	McFadden et al. 2017
*Ovabundaimpulsatilla*	ZMTAU:34891	KY442383	KY442337	KY442356	KY442419	McFadden et al. 2017
*Ovabundaobscuronata*	ZMTAU:CO 34077	KJ511307**	GU356027*	GU356004*	KJ511428**	*McFadden et al. 2011, **McFadden et al. 2014b
*Sansibiaflava*	ZMTAU:Co36004	MK400137	MK396681	MK396728	–	McFadden et al. 2019
*Sansibiaflava*	ZMTAU:Co36006	MK030486	MK030380	MK039204	–	McFadden et al. 2019
*Sansibiaflava*	ZMTAU:Co36073	MK030487	MK030381	MK039205	–	McFadden et al. 2019
*Sympodiumcaeruleum*	ZMTAU CO34185	JX203758*	JX203815*	GU356009**	KJ511430***	*McFadden and Ofwegen 2012
						**McFadden et al. 2011
						***McFadden et al. 2014b
*Xeniafisheri*	CAS:IZ:184540	KJ511311	KJ511349	KJ511389	KJ511436	McFadden et al. 2014b
*Xeniafisheri*	CAS:IZ:184541	KJ511312	KJ511350	KJ511390	KJ511437	McFadden et al. 2014b
*Xeniakusimotoensis*	CAS:IZ:184554	KJ511314	KJ511352	KJ511392	KJ511441	McFadden et al. 2014b
*Xenialepida*	CAS:IZ:184535	KJ511316	KJ511354	KJ511394	KJ511443	McFadden et al. 2014b
*Xenialepida*	CAS:IZ:184562	KJ511317	KJ511355	KJ511395	KJ511444	McFadden et al. 2014b
*Xeniamembranacea*	CAS:IZ:184536	KJ511308	KJ511345	KJ511385	KJ511432	McFadden et al. 2014b
*Xeniamembranacea*	CAS:IZ:184548	KJ511319	KJ511357	KJ511397	KJ511446	McFadden et al. 2014b
*Xeniamembranacea*	CAS:IZ:184549	KJ511320	KJ511358	KJ511398	KJ511447	McFadden et al. 2014b
*Xeniapuertogalerae*	CAS:IZ:184532	KJ511324	KJ511362	KJ511402	KJ511451	McFadden et al. 2014b
*Xeniapuertogalerae*	CAS:IZ:184539	KJ511325	KJ511363	KJ511403	KJ511452	McFadden et al. 2014b
*Xeniapuertogalerae*	CAS:IZ:184545	KJ511326	KJ511364	KJ511404	KJ511453	McFadden et al. 2014b
*Xeniaviridis*	CAS:IZ:184542	KJ511331	KJ511369	KJ511409	KJ511458	McFadden et al. 2014b
*Xeniahicksoni*	ZMTAU CO34072	JX203759*	GQ342529**	GQ342463**	KJ511438*	*McFadden and Ofwegen 2012, **Brockman and McFadden 2012
*Xeniaternatana*	CAS:IZ:184560	KJ511327	KJ511365*	KJ511405*	KJ511454	McFadden et al. 2014b
*Xeniaumbellata*	ZMTAU:36783	KY442362*	KT590452**	KT590435**	KY442431*	*McFadden et al. 2017, **Halász et al. 2019
*Xeniaumbellata*	ZMTAU:36788	KY442367*	KT590457**	KT590438**	KY442432*	*McFadden et al. 2017, **Halász et al. 2019
*Xeniaumbellata*	ZMTAU:36790	KY442369*	KT590458**	KT590439**	–	*McFadden et al. 2017, **Halász et al. 2019
*Yamazatumiubatum*	ZMTAU:Co35143	MH071864	MK030449	MK039274	–	McFadden et al. 2019
*Yamazatumiubatum*	ZMTAU:Co35144	MH071865	MH071910	MH071958	–	Benayahu et al. 2018a
*Yamazatumiubatum*	ZMTAU:Co35741	MK030452	MK030451	MH071955	–	McFadden et al. 2019
*Unomiastolonifera*	ZMTAU Co38081	MT489336	MT482554	MT487559		Benayahu et al. 2021
*Coelogorgiapalmosa*	NTM C14914	JX203698	DQ302805	GQ342413	DQ302879	McFadden et al. 2006
*Rhytismafulvum*	ZMTAU CO34124	JX203728*	GQ342478**	GQ342396**	–	*McFadden and Ofwegen 2012, **Brockman and McFadden 2012
*Paralemnaliathyrsoides*	ZMTAU:Co36976	MH516907	MH516632	MH516518	–	Benayahu et al. 2018b
*Cladielladigitulata*	MUFS-COSU14	–	–	–	LC467083	Koido et al. 2019
*Cladiellasphaerophora*	MUFS-COAK1	–	–	–	LC467084	Koido et al. 2019
*Klyxum* sp.	MUFS-COMO150	–	–	–	LC467086	Koido et al. 2019
*Klyxum* sp.	MUFS-COMO164	–	–	–	LC467087	Koido et al. 2019
*Klyxum* sp.	MUFS-COOTUD8	–	–	–	LC467088	Koido et al. 2019

## ﻿Results

### ﻿Taxonomy

#### Class Anthozoa Ehrenberg, 1831

**Subclass Octocorallia Haeckel, 1866**

**Order Alcyonacea Lamouroux, 1812**

#### Family Xeniidae Ehrenberg, 1828

##### ﻿Genus Xenia

Taxon classificationAnimaliaAlcyonaceaXeniidae

Lamarck, 1816

52A3BF13-E181-5B8C-9E48-59C7257C65CE

###### Type species.

*Xeniaumbellata* Lamarck, 1816

###### Emended diagnosis.

(Chiefly after Halász et al. 2019). Colonies are small and soft with cylindrical stalk, undivided or branched, terminating in one or more domed polyp-bearing regions. Polyps are not retractile and are always monomorphic. The dominant sclerites are ellipsoid platelets, usually abundant in all parts of the colony. They are composed of calcite rods, often dendritic or sinuous, mostly radially arranged, at least at the periphery of the sclerites. In addition to ellipsoid platelets, a few species have rods or unique spindles with pointed spear ends.

##### ﻿ Xenia konohana sp. nov.

Taxon classificationAnimaliaAlcyonaceaXeniidae

566DDCE1-F472-55BC-B6E0-211FD5C8311C

http://zoobank.org/D1BD260D-A55D-4A88-9CF6-823E06AF0504

New Japanese name: konohana-umiazami Figs 3 , 4 , 5 , 6 , 7 , 8 , 9 , 10

###### Synonym.

**Figure 2.**
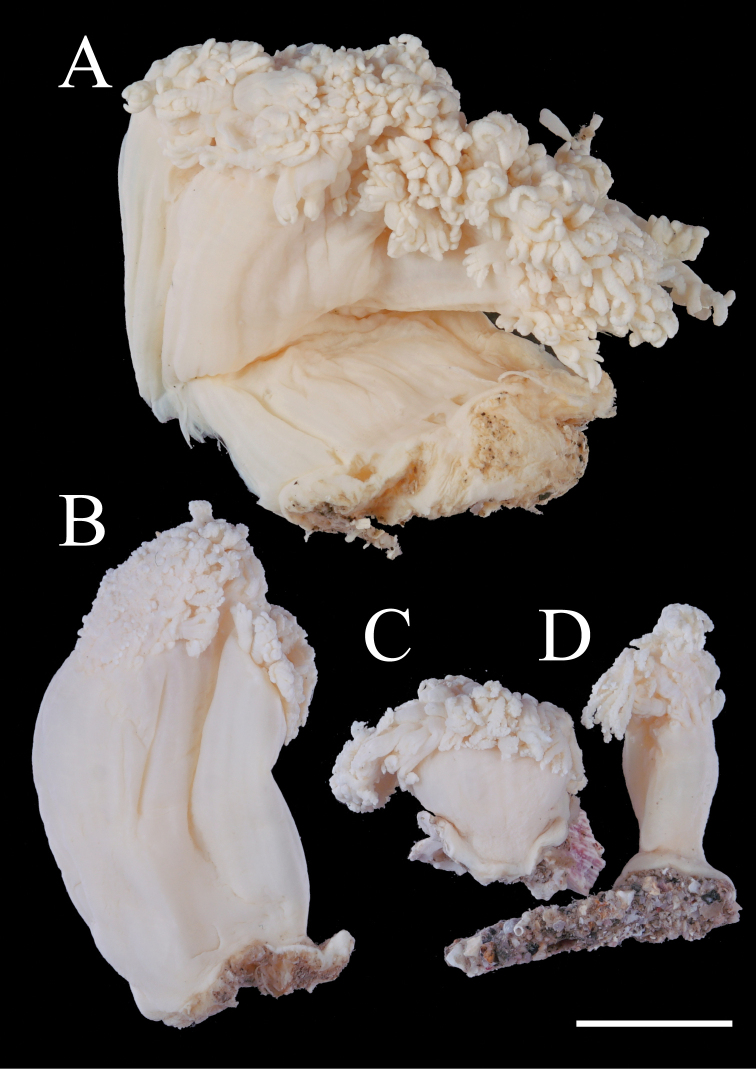
Fixed specimens of *Xeniakonohana* sp. nov. **A** holotype BF-OA-00092 **B** paratype KBF-OA-00093 **C, D** paratype KBF-OA-00094. Scale bar: 10 mm.

**Figure 3.**
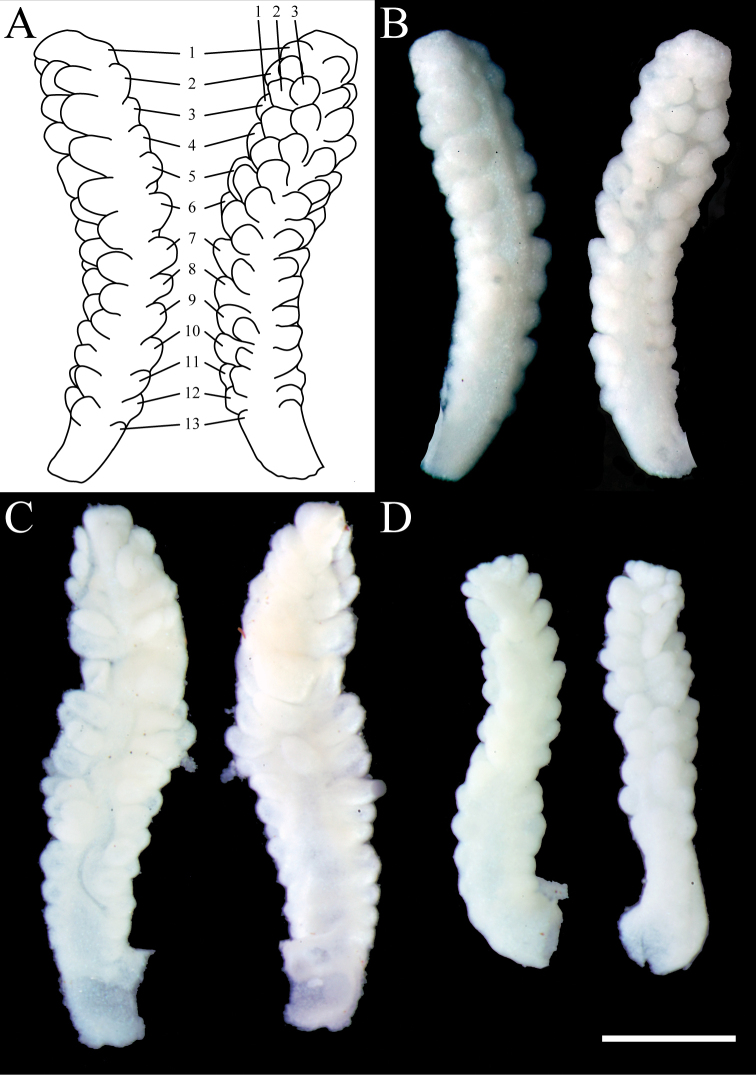
Tentacles of *Xeniakonohana* sp. nov. aboral (left) and oral sides (right) **A** schema of holotype KBF-OA-00092: three rows (the number is shown in the upper-right) and 13 pinnules at the outermost row (the number is shown in the center) **B** holotype KBF-OA-00092 **C** paratype KBF-OA-00093 **D** paratype KBF-OA-00094. Scale bar: 1 mm.

**Figure 4.**
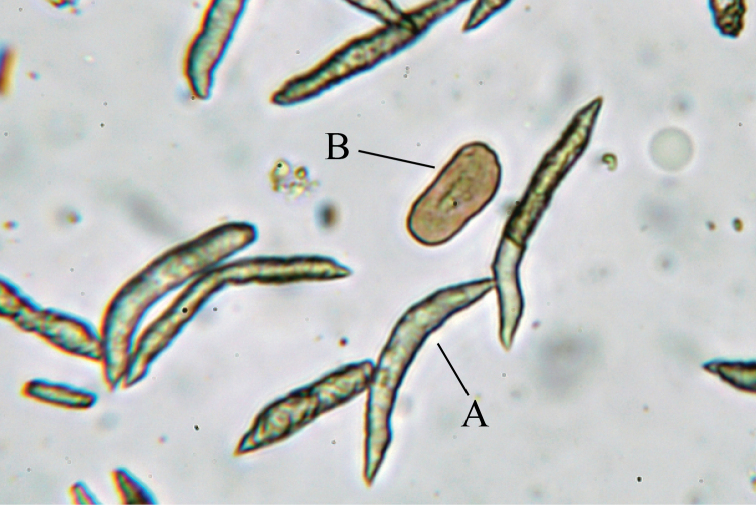
Light microscope images of sclerites in polyps of *Xeniakonohana* sp. nov., holotype KBF-OA-00092 **A** spindles **B** simple platelets.

*Xenia* sp. 1 Koido et al. 2019: Table 1, figs 2J–4J.

###### Materials.

***Holotype***: KBF-OA-00092 (MUFS-COMO4 in Koido et al. 2019), Oshima Isl., Nichinan City, Miyazaki Prefecture, depth < 5 m, July 2, 2012. ***Paratypes***: KBF-OA-00093 (MUFS-COMO53 in Koido et al. 2019), Oshima Isl., Nichinan City, Miyazaki Prefecture, depth < 10 m, December 25, 2012; KBF-OA-00094 (One colony with two stems) (MUFS-COMO54 in Koido et al. 2019), Oshima Isl., Nichinan City, Miyazaki Prefecture, depth < 10 m, December 25, 2012.

###### Descriptions.

The holotype (Fig. 2A) displays a typical *Xenia*-style growth form (Alderslade 2001; Benayahu 2010), featuring a distinct cylindrical stalk, 35 mm high and 20 mm wide attached to a rock. The colony possesses three branches 5–7 mm long from a common basal stalk. The whole colony is creamy white in ethanol. Polyps are 4.5–5.0 mm long, excluding tentacles, and 2.0 mm in diameter at their proximal part. Tentacles are 3.0–4.0 mm long and 0.3–0.5 mm wide at their proximal part.

Pinnules are arranged mostly in three rows along each side of the tentacles, leaving free median space along the oral side. This space is not always visible at the distal part of the longest tentacles. The number of rows of pinnules drops to two toward the proximal part of the tentacle, and occasionally, only a single row can be seen (Fig. 3). The outermost row usually includes 12–16 pinnules each, up to 0.23 mm long and 0.21 mm wide at the proximal part. Typically, no gap between pinnules exists, but in rare cases, a gap of approximately 0.05 mm is observed.

**Figure 5.**
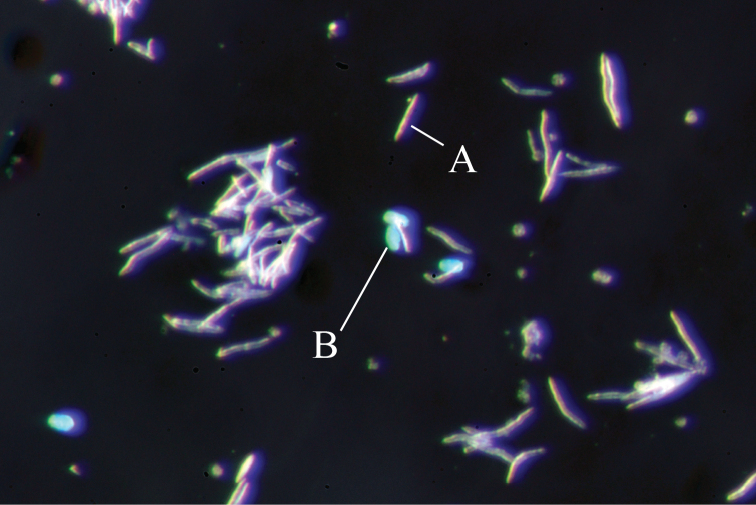
Stereoscopic microscopes images of sclerites in polyps of *Xeniakonohana* sp. nov., holotype KBF-OA-00092 **A** spindles **B** simple platelets.

Sclerites are abundant in polyps and surface layers of stalk and branches but absent interior. Under light microscopy, two forms of sclerites are observed – simple platelets (Fig. 4A) and spindles (Fig. 4B). Platelets are brown-red and spindles transparent (Fig. 4) under transmitted illumination. Platelets look pale blue and spindles appear transparent under epi-illumination (Fig. 5).

###### Polyp sclerites.

**Figure 6.**
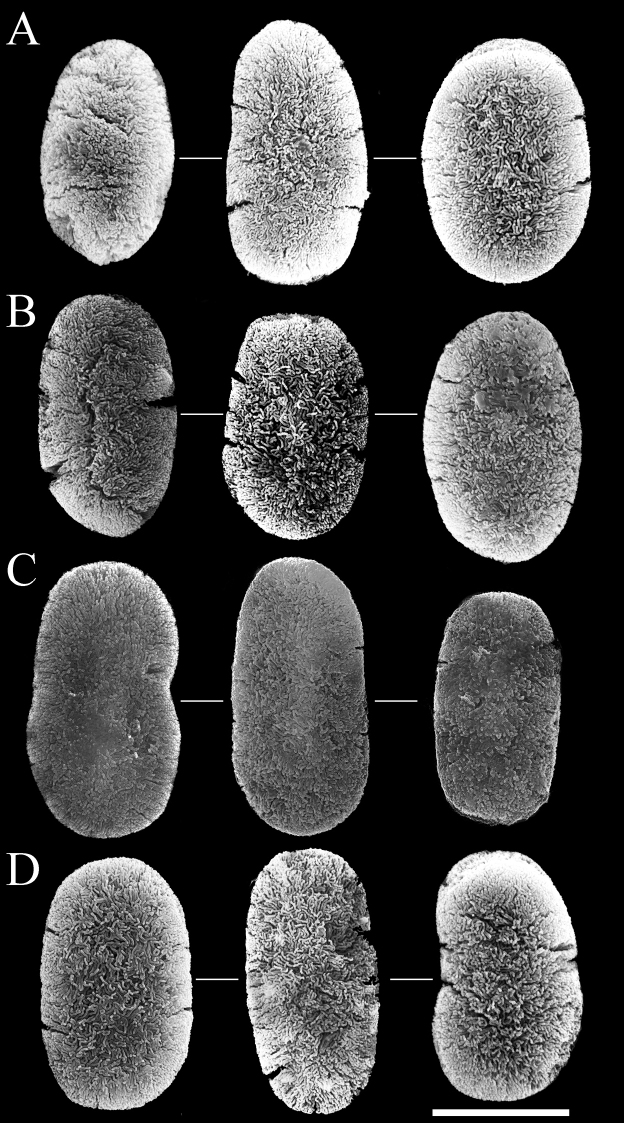
Scanning electron micrographs of platelets of *Xeniakonohana* sp. nov., holotype KBF-OA-0009 **A** in tentacles **B** in polyp body **C** in stalk surface **D** in branch surface. Scale bar: 0.010 mm.

**Figure 7.**
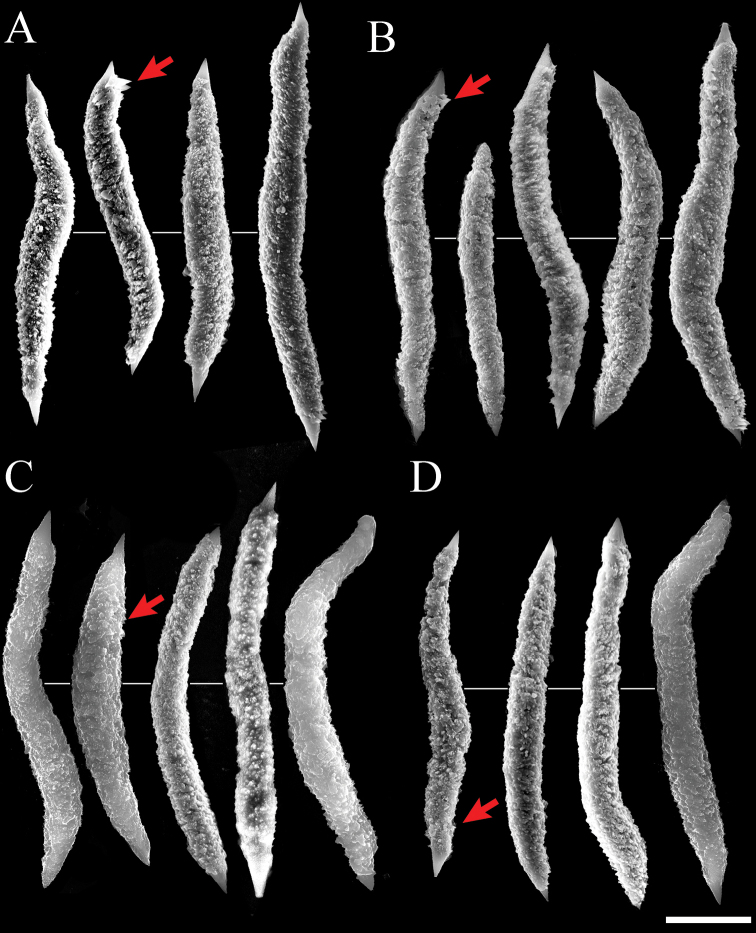
Scanning electron micrographs of spindles of *Xeniakonohana*sp. nov., holotype KBF-OA-00092 **A** in tentacles **B** in polyp body **C** in stalk surface **D** in branch surface. Arrow indicates thorns on the surface of spindles. Scale bar: 0.010 mm.

Two forms of sclerites, simple platelets and spindles, are seen in polyps (Figs 6A, B, 7A, B). Simple platelets are 0.016–0.021 mm long and 0.009–0.011 mm wide. Spindles, 0.035–0.049 mm long and 0.004–0.006 mm wide, display unique ends with pointed spear tips. Sclerite composition in tentacles (n = 124) is 7.3% simple platelets and 92.7% spindles. In the polyp body (n = 83), these proportions are 4.8% and 95.2%, respectively. Thus, the vast majority of sclerites are spindles. Some spindles have thorns on their surface.

###### Stalk and branch sclerites.

Two forms of sclerites, simple platelets and spindles, are also found in stalk and branches (Figs 6C, D, 7C, D). Simple platelets, several with an indistinct median waist, are 0.017–0.021 mm long and 0.009–0.011 mm wide. Spindles are 0.038–0.049 mm long and 0.004–0.006 mm wide. All spindles are more or less bent. Sclerite composition in stalk (n = 104) is 7.7% simple platelets and 92.3% spindles. Thus, the vast majority of sclerites are spindles.

###### Microstructure of sclerites.

**Figure 8.**
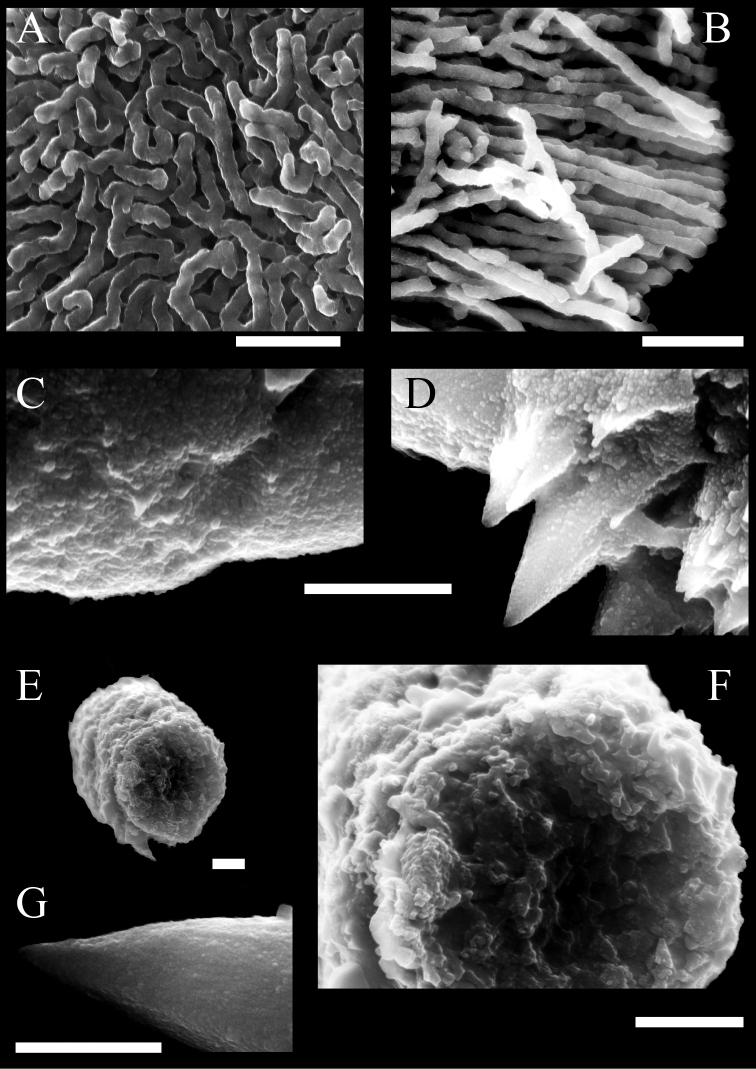
Scanning electron micrographs of the surface of sclerites in tentacles of *Xeniakonohana* sp. nov., holotype KBF-OA-00092 **A** surface of platelets covered by minute papillae **B** broken platelets with radial dendritic rods **C** central surface of spindle covered by minute granular **D** thorns on the surface of spindles **E** broken spindle **F** close-up view of a broken spindle with fused grain **G** tip of a spindle. Scale bar: 0.001 mm.

The platelets are composed of branched sinuous dendritic rods within the sclerite interior. SEM at 30,000–50,000× magnification shows distal parts of rods that line up almost vertically and parallel to the surface (Fig. 8A, B). The spindles are composed of fused grains with a granular appearance (Fig. 8C, D). Fused grains also exist inside, which can be observed in cross-sections of broken spindles (Fig. 8E, F). Both ends of the spindles are relatively smooth (Fig. 8G). Thorns may form on the surface of spindles (Fig. 7, red arrows indicate the thorn, Fig. 8D shows the thorn expansion).

###### Variation.

**Figure 9.**
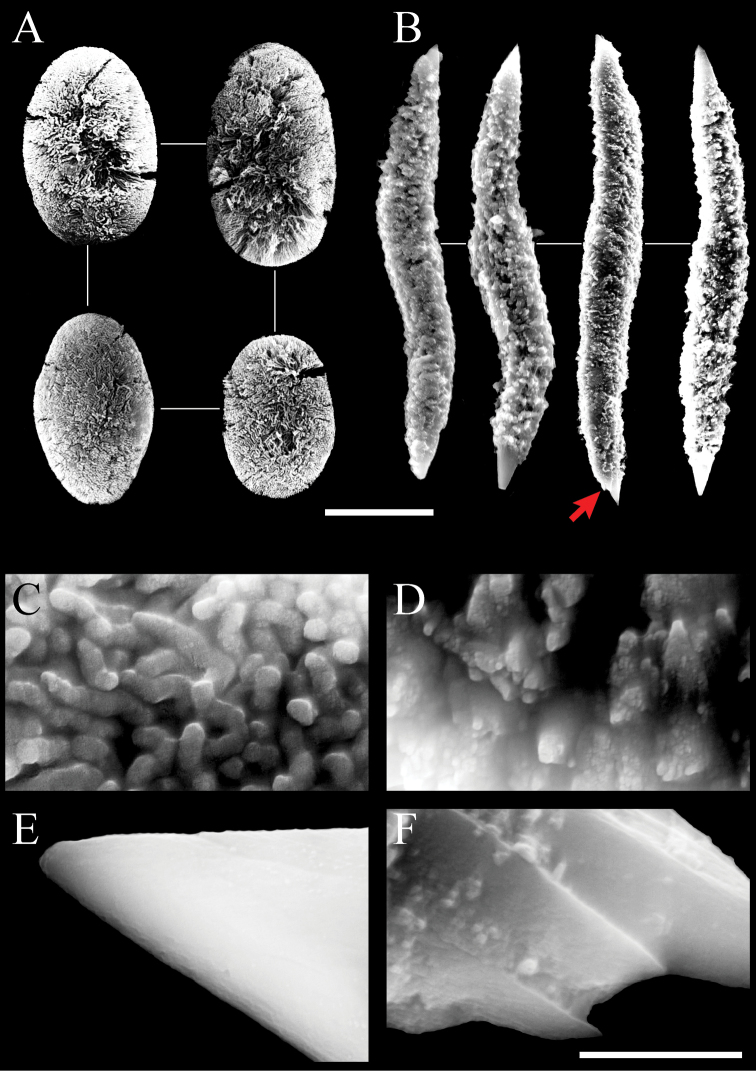
Scanning electron micrographs of paratype (KBF-OA-00093) of *Xeniakonohana* sp. nov.: **A** platelets **B** spindles (arrow indicates thorns on the surface of spindles) **C** surface of platelets **D** central surface of spindle **E** tip surface of a spindle **F** thorns on the surface of spindles. Scale bar: 0.01 mm (**A, B**); 0.001 mm (**C–F**).

**Figure 10.**
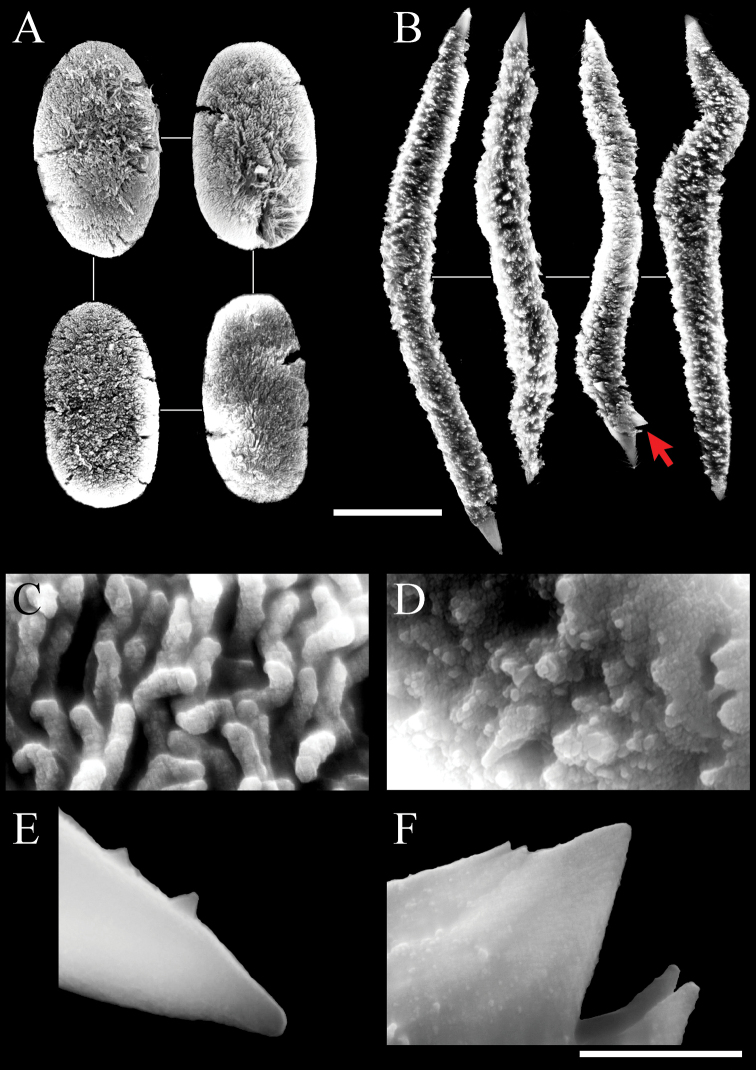
Scanning electron micrographs of paratype (KBF-OA-00094) of *Xeniakonohana* sp. nov.: **A** platelets **B** spindles (arrow indicates thorns on the surface of spindles) **C** surface of platelets **D** central surface of spindle **E** tip surface of a spindle **F** thorns on the surface of spindles. Scale bar: 0.01 mm (**A, B**); 0.001 mm (**C–F**).

Two preserved paratypes (KBF-OA-00093, KBF-OA-00094) differ in size (Fig. 2B, C). Both paratypes are smaller than the holotype (30 mm high, 15 mm wide of KBF-OA-00093, and 9–16 mm high, 6–9 mm wide of KBF-OA-00094). One paratype (KBF-OA-00094) does not branch but has two stalks connected at the bottom, although this specimen, accidentally, is broken into two pieces (Fig. 2C, D). Tentacle size is 4.0 mm long and 0.5 mm wide for KBF-OA-00093 and 3.0 mm long and 0.5 mm wide for KBF-OA-00094 (Fig. 3C, D). Paratypes display three rows of pinnules along each side of tentacles, consistent with the holotype. Pinnule numbers in the outermost row are 13–16 for KBF-OA-00093, and 12–14 for KBF-OA-00094, compared to 12–16 for the holotype. All paratypes have the two forms of sclerites as well as holotype (Fig. 9, 10), and are similar in the composition. In all parts of all specimens, the vast majority of sclerites are spindles, with the percentages being approximately 83–94% (Table 2).

**Table 2.** Sclerite composition of *Xeniakonohana* sp. nov.

		Tentacles		Polyp body		Stalk	
		platelets	spindles	platelets	spindles	platelets	spindles
KBF-OA-00092 (holotype)	Fig. 2A	n = 124		n = 83		n = 104	
		7.3%	92.7%	4.8%	95.2%	7.7%	92.3%
KBF-OA-00093 (paratype)	Fig. 2B	n = 123		n = 132		n = 85	
		5.7%	94.3%	10.6%	89.4%	7.1%	92.9%
KBF-OA-00094 (paratype)	Fig. 2C	n = 138		n = 103		n = 91	
		10.1%	89.9%	5.8%	94.2%	6.6%	93.4%
	Fig. 2D	n = 92		n = 152		n = 96	
		12.0%	88.0%	17.1%	82.9%	7.3%	92.7%

###### Locality.

The species is common in waters around Oshima Island, Miyazaki, Japan, at depths from 5 to 10 m. Specimens exist attached to the surface of rocks or rock debris.

###### Etymology.

Konohana is named after a goddess in Japanese mythology, “Konohanasakuya-hime” (“hime” is “princess” in English). Her shrine is in Miyazaki Prefecture. The present study also proposes a standard Japanese name “konohana-umiazami” for *X.konohana* sp. nov. The specimen KBF-OA-00092 is designated as the standard specimen for this new Japanese name.

###### Remarks.

Most *Xenia* species have only ellipsoid platelets or spheroid sclerites (Halász et al. 2019). Although only two species, *X.membranacea* Schenk, 1896 and *X.depressa* Kükenthal, 1909 have been reported to display rod-shaped sclerites in their original descriptions, this type of sclerite has not been found in the syntype of *X.membranacea* (Halász et al. 2019), and *X.depressa* has never been re-described and the existence of the type materials are unknown. Therefore, we treated the existence of rod-shaped sclerites as either incorrect for *X.membranacea* or unverified for *X.depressa* in this study. On the other hand, *X.konohana* sp. nov. (= *Xenia* sp. 1 by Koido et al. 2019) has unique spindle sclerites in addition to ellipsoid platelets (Figs 4–10). This combination does not occur in other species in the genus. Moreover, it is clear that spindles are the majority sclerites in tentacles, polyp body and stalks for all three specimens (KBF-OA-00092 to KBF-OA-00094).

All three specimens (KBF-OA-00092 to KBF-OA-00094) were nearly identical in sclerite shape, size and composition of two types of sclerite forms (xeniid platelets and unique spindles), number of pinnules, and molecular phylogenetic position. Eight species of *Xenia* (*X.blumi* Schenk, 1896, *X.crassa* Schenk, 1896, *X.cylindrica*Roxas 1933, *X.fisheri* Roxas, 1933, *X.garciae* Bourne, 1895, *X.hicksoni* Ashworth, 1899, *X.ternatana* Schenk, 1896, and *X.viridis* Schenk, 1896), which partly overlap with *X.konohana* sp. nov. in exhibiting platelet sclerites, 3–4 rows of pinnules and 12–23 outermost row of pinnules, are distinguishable by the absence of the specific sclerite form, “unique spindle” (Table 3). A variation of pinnules has been reported in many species in xeniid genera, and the number of pinnules is likely to be unreliable as a character to determine the species boundaries (Halász et al. 2019; McFadden et al. 2017). Therefore, the information on sclerites is more important than ever as a character for identifying species boundaries.

**Table 3.** Morphological comparison with congeneric species. *including oval, round, circles, discs, and biscuit-like shapes. Dashes means absent. Question marks mean unverified. NR means not reported. Note that morphological data were referred from the re-description paper by Halász et al. (2019) rather than the original descriptions for some species.

Species	Rows of pinnules	Pinnules in the outermost row	Sclerites			Crest on the sclerites	Main branch	Secondary branches	References
			platelets*	rods	Spindles				
*X.bauiana*	4	26–30	present	–	–	–	NR	NR	Halász et al. 2019
*X.blumi*	3	18–20	present	–	–	–	NR	NR	Halász et al. 2019
*X.crassa*	3–4	13–18	present	–	–	present	NR	NR	Halász et al. 2019
*X.cylindricacy*	3	18–20	present	–	–	NR	2	–	Roxas 1933
*X.depressa*	2	18–26	present	?	–	NR	NR	NR	Kükenthal 1909
*X.delicata*	3–4	18–23	–	–	–	–	0–5	0–3	Halász et al. 2019
*X.elongata*	3–4	20–24	present	–	–	NR	2–3	–	Dana 1846, Imahara 1992
*X.fimbriata*	3	8–15	–	–	–	NR	2–3	present	Utinomi 1955
*X.fisheri*	3	18–22	present	–	–	NR	–	–	Roxas 1933
*X.flexibilis*	4	14–32	present	–	–	–	NR	NR	Halász et al. 2019
*X.fusca*	4(3–5)	14–22	present	–	–	–	NR	NR	Halász et al. 2019
*X.garciae*	3	16–22	present	–	–	present	–	–	Halász et al. 2019
*X.grasshoffi*	4	15–24	present	–	–	present	NR	NR	Halász et al. 2019
*X.hicksoni*	3	12–20	present	–	–	NR	usually branched	2	Ashworth 1899,
									Utinomi 1950
*X.kuekenthali*	1	8–10	–	–	–	–	5	0–2	Halász et al. 2019
*X.kusimotoensis*	2	10–12	present	–	–	NR	2	–	Utinomi 1955
*X.lepida*	3	28–34	–	–	–	–	present	3^rd^ branches	Halász et al. 2019
*X.mayi*	5	24–32	present	–	–	NR	single or divided	–	Roxas 1933
*X.membranacea*	4	20–25	present	–	–	present	8	NR	Halász et al. 2019
*X.multipinnata*	3–4	40–50	–	–	–	NR	present	–	Tixier-Durivault 1966
*X.multispiculata*	2–3	26–30	present	–	–	NR	present	–	Kükenthal 1909
*X.mucosa*	4	30–42	–	–	–	–	2	0–2	Halász et al. 2019
*X.novaebritanniae*	2	9–10	present	–	–	–	NR	NR	Halász et al. 2019
*X.rubens*	4(3–5)	12–19	present	–	–	–	2	–	Halász et al. 2019
*X.sansibariana*	4	26–33	–	–	–	–	NR	NR	Halász et al. 2019
*X.stellifera*	4–9	<9	present	–	–	NR	present	present	Verseveldt 1977
*X.ternatana*	3	15–23	present	–	–	present	NR	NR	Halász et al. 2020
*X.tripartita*	3	5–6	present	–	–	NR	–	–	Roxas 1933
*X.tumbatuana*	3	NR	–	–	–	NR	present	–	May 1898
*X.umbellata*	3	19–22	present	–	–	–	–	–	Halász et al. 2019
*X.viridis*	3	15–22	present	–	–	present	NR	NR	Halász et al. 2019
***X.konohana* sp. nov.**	**3**	**12–18**	**present**	–	**Present**	–	**2–3**	–	**This study**

### ﻿Molecular phylogenetic results

**Figure 11.**
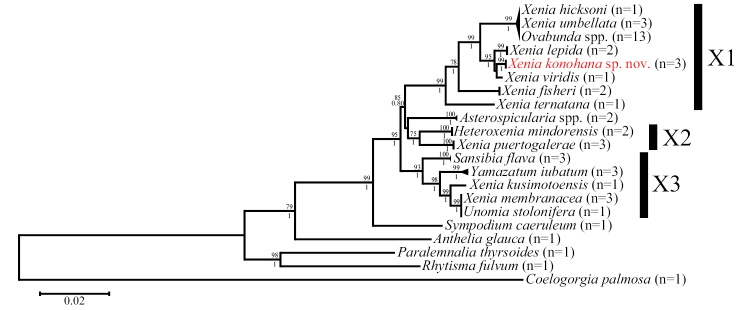
Phylogenetic relationships of species in the Xeniidae based on the concatenated *mtMutS, COI* and *28S* sequences. Numbers above main branches show percentages of bootstrap values (> 50%) in maximum likelihood analysis; numbers below main branches show Bayesian posterior probabilities. X1, X2 and X3 denote clades defined by McFadden et al. (2014b). *Xeniakonohana* sp. nov. is shown in red.

**Figure 12.**
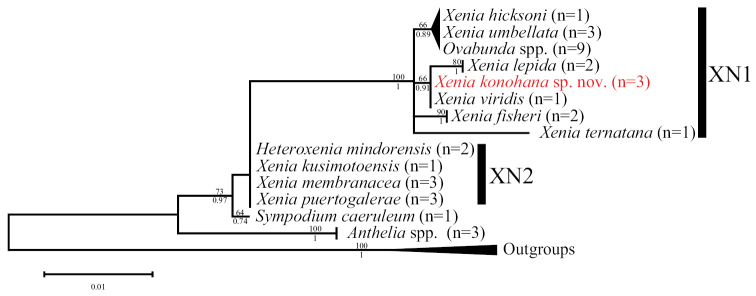
Phylogenetic relationships of species in the Xeniidae based on *ND2* sequences. Numbers above main branches show percentages of bootstrap values (> 50%) in maximum likelihood analysis; numbers below main branches show Bayesian posterior probabilities. *Xeniakonohana* sp. nov. is shown in red.

Molecular phylogenetic trees using the ML and Bayes methods showed very similar topologies. Therefore, in this study, only ML trees are shown (Figs 11, 12). As pointed out in previous studies (Halász et al. 2019; Benayahu et al. 2021), the genus *Xenia* is paraphyletic and polyphyletic with some other taxa, and separated into three clades (clades X1–X3) in the *mtMutS+COI*+*28S* tree (Fig. 11). All three clades were supported by high bootstrap values (75 to 99%) and posterior probabilities (1). Asides from *Xenia*, clade X1 included *Ovabunda*, clade X2 included *Heteroxenia*, and clade X3 included *Sansibia*, *Yamazatum* and *Unomia*. All three specimens of *X.konohana* sp. nov., which had the same DNA sequences for all four markers, belonged to clade X1 forming a sister clade with *Ovabunda* spp., *X.umbellata* and *X.hicksoni*, ​and united with *X.lepida* Verseveldt, 1971 and *X.viridis* within a strongly supported subclade (bootstrap values: 95%, posterior probability: 1).

On the other hand, in the *ND2* tree, *Xenia* was separated into only two clades (XN1 and XN2) (Fig. 12). Clade XN1 was strongly supported by high bootstrap value (100%) and posterior probability (1), and included the same members with all three specimens of *X.konohana* sp. nov. in clade X1 in the *mtMutS+COI*+*28S* tree. For clade XN2, this clade was not supported by bootstrap values and posterior probabilities, but three *Xenia* species and *Heteroxeniamindorensis* in this clade were genetically identical. Clade XN2 included members belonging to both clades X2 and X3 in the *mtMutS+COI*+*28S* tree.

Although *X.viridis* was not genetically separated from *X.konohana* sp. nov. in the *ND2* tree (Fig. 12), they were clearly separated from each other in the *mtMutS+COI*+*28S* tree (Fig. 11). Thus, the molecular phylogenetic tree based on the concatenated DNA sequences of *mtMutS*, *COI*, and *28S*, and the tree based on *ND2* support the phylogenetic position of *X.konohana* sp. nov. in the genus *Xenia* (Figs 11, 12).

## ﻿Discussion

The genus *Xenia* is polyphyletic and paraphyletic with other xeniid genera such as *Ovabunda*, *Heteroxenia*, *Sansibia*, *Asterospicularia*, *Unomia*, and *Yamazatum* based on molecular studies (Janes et al. 2014; McFadden et al. 2014b; Benayahu et al. 2018a; Halász et al. 2019; Benayahu et al. 2021). In the present study, *Xenia* was also polyphyletic as well as paraphyletic with some other genera (Figs 11, 12), but *X.konohana* sp. nov. formed a clade with two congeneric species, *X.lepida* and *X.viridis*, and was closely related to a sister clade with *Ovabunda* spp., *X.hicksoni*, and *X.umbellata*. These four *Xenia* species are similar to *X.konohana* sp. nov. in the number of rows and the outermost row of pinnules, but they do not exhibit spindle sclerites. *Ovabunda* exhibits only simple platelets like *Xenia*, but it also displays a corpuscular surface microstructure on platelet surfaces. *Xenia*, including *X.konohana* sp. nov., exhibits a dendritic microstructure on these surfaces of simple platelets. Further taxonomic revision of *Xenia* and related genera such as *Ovabunda*, *Heteroxenia*, *Sansibia*, *Asterospicularia*, *Unomia*, *and Yamazatum* may be necessary due to these phylogenetic relationships. Still, we conclude that *Xeniakonohana* sp. nov. is a new member of *Xenia* based on molecular phylogenetic relationships and the presence of unique spindles along with *Xenia*-specific ellipsoid platelets with dendritic surface microstructure.

## Supplementary Material

XML Treatment for Xenia

XML Treatment for Xenia konohana
